# Partial nephrectomy for a Bosniak IV cystic renal mass mimicking a simple renal cyst adjacent to a solid renal tumor

**DOI:** 10.1002/iju5.12227

**Published:** 2020-10-10

**Authors:** Toshiki Oka, Koji Hatano, Yohei Okuda, Toshihisa Asakura, Yasutomo Nakai, Masashi Nakayama, Ken‐ichi Kakimoto, Chiaki Kubo, Shin‐ichi Nakatsuka, Kazuo Nishimura

**Affiliations:** ^1^ Departments of Department of Urology Pathology and Cytology Osaka Japan; ^2^ Department of Pathology and Cytology Osaka International Cancer Institute Osaka Japan

**Keywords:** Bosniak classification, cyst wall, partial nephrectomy, renal cell carcinoma, simple renal cyst

## Abstract

**Introduction:**

Renal tumors are often associated with renal cysts. Meanwhile, in some cases there are challenging issues of how to diagnose renal cystic tumors and to decide surgical procedures.

**Case presentation:**

A 75‐year‐old man was referred to our department for a 21‐mm tumor by his left kidney. Contrast‐enhanced computed tomography showed an intense contrast uptake the tumor, which was adjacent to a 64‐mm unilocular renal cyst with no contrasting effects. It was clinically diagnosed as renal cell carcinoma, stage T1aN0M0, and treated with robot‐assisted partial nephrectomy, for both the solid tumor and the adjacent cyst. Pathological findings revealed a tumor cell clump within the cyst wall, concurrent with the renal cell carcinoma. The patient has remained free of disease at 1 year after surgery.

**Conclusion:**

A partial nephrectomy that includes the entire cyst wall should be considered for renal tumor associated with unilocular renal cyst.

**Abbreviations & Acronyms**
CT: computed tomography
RAPN: robot‐assisted partial nephrectomy
RCC: renal cell carcinoma
SRC: simple renal cyst

**Keynote message**

Some renal tumors associated with renal cysts can pose a diagnostic and therapeutic dilemma. This case report provides a valuable insight into the surgical procedure of a Bosniak IV lesion mimicking a SRC adjacent to a solid renal tumor. Unilocular renal cysts associated with RCC may harbor tumor cells within the cyst wall.

## Introduction

SRC is a common benign kidney lesion. Its prevalence increases with age; reportedly, 23.9% of people aged 60 years or older have SRC.^1^ Bosniak classification for renal cystic masses is widely utilized to predict cystic tumor grades and guide decisions regarding surgery or follow‐up.^2^ Bosniak category I and category II lesions are simple and minimally complex (respectively) cysts that require no further work‐up.^3^ However, several reports have shown RCC to originate from SRCs.^4^, ^5^

Here, we describe a case of a Bosniak IV lesion mimicking a SRC adjacent to a solid renal tumor. Partial nephrectomy is usually indicated for a small RCC, but where to resect a tumor associated with unilocular renal cysts warrants some reflection.

## Case presentation

**Fig. 1:**
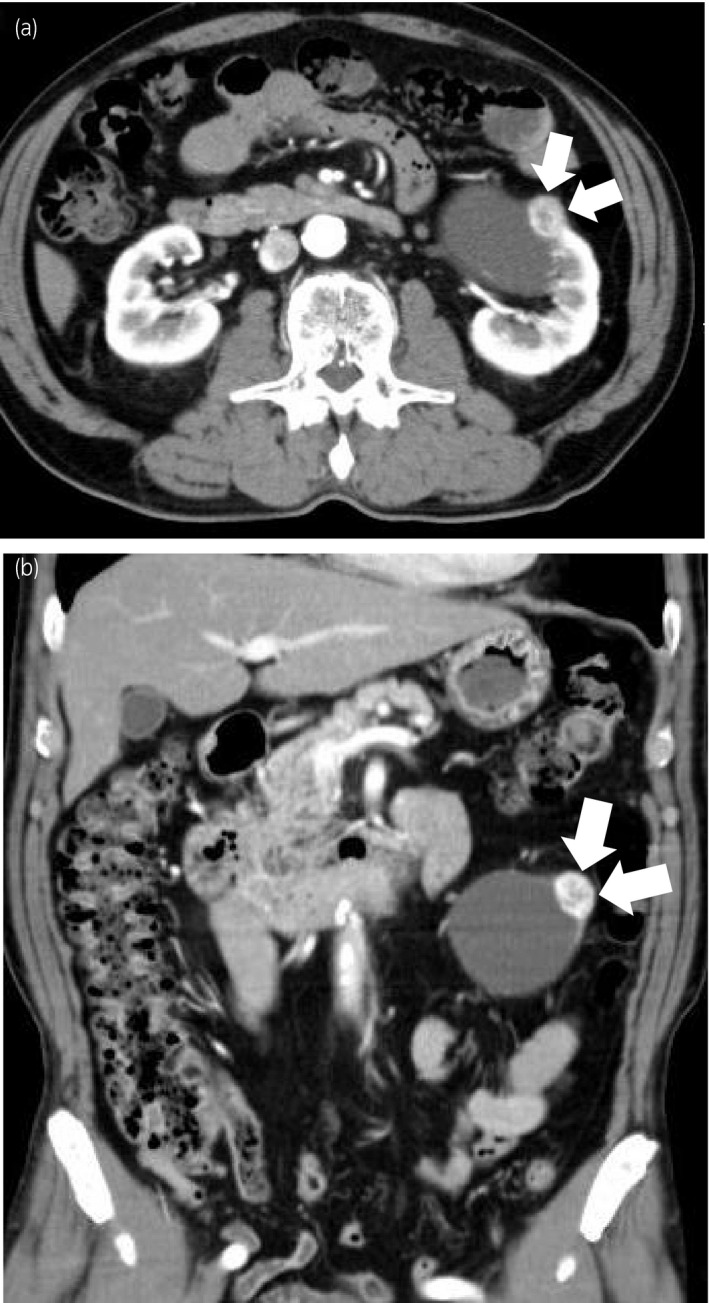
Contrast‐enhanced CT showed an enhanced tumor adjacent to a renal cyst in the left kidney. Arrows: tumor. (a) Axial section. (b) Coronal section.

A 75‐year‐old man was referred to our department because of an enhanced renal mass which was detected by contrast‐enhanced abdominal CT. The CT showed a 64‐mm unilocular cyst in the lower pole of the left kidney, with a 21‐mm mass within the cyst. The mass showed an intense contrast uptake in the corticomedullary phase and typical washout in the nephrographic phase. The CT showed no wall thickening or contrasting effects in the renal cyst wall (Fig. 1). The lesion was clinically diagnosed as RCC stage T1aN0M0, and treated with a RAPN, using a transperitoneal approach. Because during surgery, the wall of the renal cyst was found to be thickened, we decided to completely resect the renal cyst and tumor. We performed a fine needle aspiration of the renal cyst prior to tumor excision to avoid wounding the cyst. The cyst contained blackish green fluid which was negative for cytological diagnosis. The entire cyst and tumor were resected from renal parenchyma sharply and bluntly (surgery: 193 min; robotic console use: 159 min; ischemia: 13 min; small amount of blood loss).

### Surgical specimen and histopathology

**Fig. 2:**
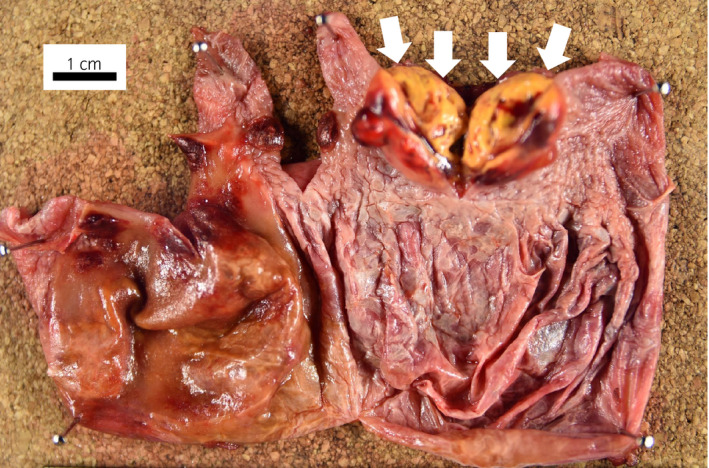
Macroscopic surgical specimen showing renal tumor in the renal cyst. Arrows: tumor.

**Fig. 3:**
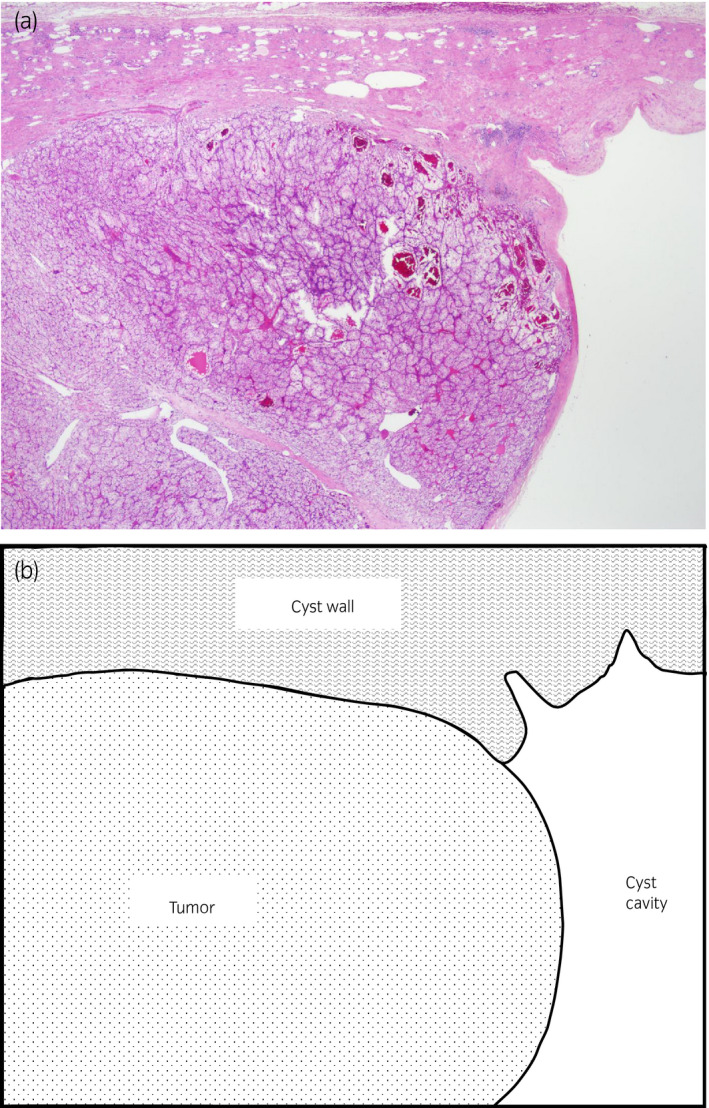
Microscopic appearance of the lesion showing clear‐cell RCC arising from the renal cyst. (a) Hematoxylin and eosin stain, (b) Schematic representation.

**Fig. 4:**
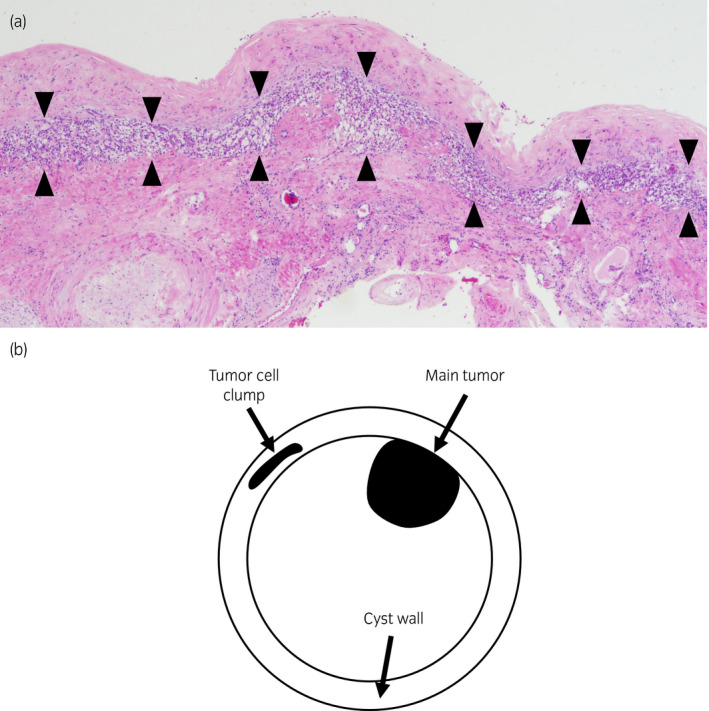
(a) Pathological examination of the cyst wall revealed clumps of clear‐cell type tumor cells in part of the cyst wall, away from the tumor. Arrows: tumor cells. (b) Schema shows positioning of main tumor and tumor cell clump.

A yellow tumor nodule (17 × 17 × 15 mm) was found in the unilocular cyst, with a moderately thick wall (Fig. 2). The tumor showed alveolar or cystic architecture by tumor cells with clear cytoplasm. The pathological findings were compatible with clear‐cell RCC, grade 2 (World Health Organization/International Society of Urological Pathology grade) with no lymphovascular invasion, pT1a (Fig. 3a,b). A cluster of tumor cells with the same morphology was found within the cyst wall, apart from the main tumor (Fig. 4a,b). The cyst wall adjacent to the renal parenchyma was lined with tubular renal cells with no atypia.

The patient has remained free of disease at 1 year after surgery.

## Discussion

Bosniak classification of ≥III indicates likely malignancy,^6^ and that surgery is therefore indicated. In our case, although the enhanced renal mass was a clear candidate for resection which was a characteristic of Bosniak IV lesion, removing the entire cystic lesion was a less obvious decision.

A recent meta‐analysis showed malignancy rates by Bosniak category I, II, and IIF were 3.2%, 6.0%, and 3.7%, respectively.^3^ Sakai *et al*.^4^ reported a patient who developed cystic renal carcinoma from a SRC over a course of 6 years. Hartman *et al*.^7^ proposed four categories for cystic renal tumor: (1) intrinsic cystic growth as a multiloculated fluid‐filled mass; (2) intrinsic cystic growth as a unilocular fluid‐filled mass; (3) cystic necrosis; and (4) origin from the epithelium of a preexisting SRC. Our case corresponds to category (4). Lai *et al*.^5^ reported two cases of RCC originating in free walls of SRCs, which were incidentally diagnosed after unroofing the SRCs. In addition, they reviewed 14 cases of RCC that originated from SRCs, including their own reported cases.^5^ All 14 cases were classified as Bosniak category I or II. Therefore, even if contrast‐enhanced CT indicates a SRC, the SRC may already have a tumor precursor lesion that would appear in a future image. When RCC is associated with SRC, complete resection of the cyst wall should be considered, as the cyst wall may harbor underlying tumor cells.

Both clear‐cell RCC and papillary RCC reportedly originate from renal proximal tubules.^8^ Kragel *et al*.^9^ analyzed the origins of both tumor types, using immunohistochemical markers and showed that atypical renal cysts and RCC predominantly arose from proximal tubules, whereas SRCs predominantly arose from distal tubules. Based on these findings, the histological transition of SRC to atypical renal cysts or RCC is unclear. Nanpo *et al*.^10^ reported a case of carcinoma in a collecting duct that arose from a renal cyst wall. In this case, a diverticulum was formed by weakening the collecting‐tubule basement membrane, in which a SRC was thought to be formed.^11^, ^12^

Cystic renal carcinoma generally grows slowly and has characteristics of low‐grade malignancy.^13^, ^14^ Therefore, surgery for cystic renal carcinoma should aim for renal preservation where possible. RAPN has become the mainstay procedure for kidney preservation surgery. Novara *et al*.^15^ performed RAPN for 54 cystic renal carcinomas and reported that perioperative and pathological outcomes were equivalent to those for solid renal carcinoma. In surgery for cystic renal tumors, inadvertent cyst rupture and tumor spillage should be avoided. Although the risk of dissemination owing to intraoperative cyst rupture is controversial,^16^, ^17^ Santiago *et al*.^18^ and Limb *et al*.^19^ reported that intraoperative cyst puncture did not affect oncological outcomes. Meanwhile, Hayakawa *et al*.^20^ reported that the cyst fluid cytology in cystic RCC had a positive rate of 16.7%. When renal tumors are associated with large SRCs, cyst puncture prior to partial nephrectomy is an option, but it is necessary to confirm that the cyst fluid contains no tumor cells. In addition, the patient should be followed‐up for local recurrence and metastasis over a long time.

## Conclusion

Partial nephrectomy that includes the entire cyst wall should be considered for renal tumors associated with unilocular cysts even if preoperative image indicates SRCs. There is a potential risk of harboring tumor cells within the cyst walls.

## Conflict of interest

The authors declare no conflict of interest.
